# ‘This is not what I want for my children’: agency and parenting in Danish asylum centres

**DOI:** 10.1093/eurpub/ckac036

**Published:** 2022-04-25

**Authors:** Amina Barghadouch, Morten Skovdal, Marie Norredam, Kathrine Vitus

**Affiliations:** 1 Research Centre for Migration, Ethnicity and Health (MESU), Section for Health Services Research, Department of Public Health, University of Copenhagen, Copenhagen, Denmark; 2 Department of Sociology and Social Work, The Faculty of Social Sciences, Aalborg University, Copenhagen, Denmark

## Abstract

**Background:**

Children of asylum-seeking families constitute a particularly vulnerable group, and there is growing interest in understanding how asylum-seeking parents can be supported to safeguard the health, wellbeing and growth of their children. In this study, we examine the capabilities of asylum-seeking parents to act on the support and advice provided by child health nurses in Danish asylum centres.

**Methods:**

We draw on semi-structured qualitative interviews with 11 asylum-seeking families (corresponding to 15 parents) living in two asylum centres run by the Danish Red Cross.

**Results:**

The findings illustrate that asylum-seeking parents’ agentic capabilities to take care of their children are tightly constrained by their housing constrictions and living conditions, insufficient money allowances, regular relocations and juridical status as asylum-seekers. These physical and organizational structures and the pervasive uncertainty related to being asylum-seekers inhibited parents from acting on advice from child health nurses, and from providing their children with a safe, healthy and stable environment.

**Conclusions:**

Asylum-seeking parents face the task of taking care of their children within particular physical, organizational and juridical structures, which tightly constrain their ability to parent their children well, or to follow advice provided by child health nurses.

## Introduction

Parenting involves flexible capabilities to ensure children’s physical, emotional, psychosocial and intellectual developmental needs, over time and across different contexts.^1^ Asylum-seeking children are especially in need of supportive parents.^2^ However, involuntary migration, and an asylum-seeking process in a new and unfamiliar country, can have an adverse influence on parental autonomy over decision-making in their own lives.^3^ In Denmark, the day-to-day administrators of asylum centres are obliged to support asylum-seeking parents’ efforts to take responsibility for their own and their children’s health and wellbeing.^4^ What affects the capabilities of asylum-seeking parents to heed the advice and take advantage of the support provided by child health nurses (CHNs) in Danish asylum centres in carrying out their parenting role? We set out to explore this question, both to draw attention to the incapacitating roles of the physical, organizational and juridical structures of the asylum system, and to highlight the inevitable contradictions within it. We introduce the notion of ‘bounded agency’ to show how these structures undermine parenting capabilities.

### The organization of the asylum-seeking process

It is common practice worldwide for countries that receive asylum seekers, including European countries, to have established reception facilities for these asylum-seekers: from their arrival, throughout their asylum-seeking process and until a decision of their application for asylum has been made.^5^ In Denmark, asylum centres are governed by the Danish Immigration Service (DIS), which subcontracts daily housing and care services within the centres to the Danish Red Cross (DRC) and municipalities. Asylum-seekers receive basic support, including housing, food/money allowance and necessary healthcare from the operators of asylum centres. An asylum-seeking process in Denmark begins in ‘Phase One’, where asylum-seekers arrive in the DRC reception centre, are registered by the police, interviewed by the DIS and offered a medical check-up in the DRC health clinic.^6^ In ‘Phase Two’, the asylum application is processed while asylum-seekers live in regular asylum centres (run by the DRC or Danish municipalities). If the DIS initially declines the asylum application, an appeal is forwarded automatically to the Refugee Appeals Board, and asylum-seekers are still considered to be in Phase Two. Phase Two may last for several years, where it is also common to be moved between different centres. This ‘waiting phase’ has been associated with uncertainty for many families.^7^ If the asylum application is finally declined, asylum-seekers enter ‘Phase Three’ and move to a departure centre or if accepted, to 1 of 98 municipalities in charge of the refugee integration process.

### Being an asylum-seeking parent

An emerging body of evidence indicates that structures within the asylum system and the asylum-seeking process negatively affect the parenting role of asylum-seekers. Studies across Europe have identified that uncertainty about the future, and the long processing times of the asylum application may cause parental distress, which also affects children in the family.^2^^,^^7–9^ Little is however known about the enabling capacity of asylum systems to support asylum-seekers’ parenting efforts. Within Danish asylum centres run by the DRC, families with children are offered enrolment in a child health programme. This includes regular consultations by DRC CHNs, where parents are introduced to childcare support, including monitoring of their children’s growth, nutrition, general wellbeing and vaccinations.^6^ We have elsewhere reported on how CHNs form deep and caring relationships with asylum-seeking parents to achieve this.^10^ An explicit aim of the programme is to equip, support and encourage asylum-seekers to take care of their children. However, we need to understand whether parents also feel capable of acting on CHNs’ advice, and how the asylum structure and process affect their practical ability to do so.

To explore and understand this further, we draw on the concept of ‘bounded agency’, which understands individuals as actors within structures with space open for ‘some’ action, yet within the constraints of a ‘social landscape’.^11^ A focus on the bounded agency of asylum-seeking parents in DRC asylum centres offers insight into the inevitable contradictions in how parents are, on the one hand, encouraged and supported by CHNs to have, and practise, parental agency, and on the other, are bounded by the asylum-seeking ‘landscape’ in enacting this advice. These insights are crucial to inform healthcare, immigration and social services professionals and policies in countries that receive asylum-seekers, as these play important roles in protecting and supporting asylum-seeking parents’ agentic capabilities.

## Aim

The aim of this study is to examine the capabilities of asylum-seeking parents to act on the support provided by CHNs within asylum centres, as part of their daily parenting efforts.

## Methods

We used a qualitative study approach, conducted by semi-structured interviews with asylum-seeking parents who lived in two regular DRC-run asylum centres. This design enabled insight into the perspectives, motives and beliefs^12^ of asylum-seeking parents, which were crucial to understanding their experiences of taking care of their children. The interviews were conducted by A.B. from January to March 2018, as part of a qualitative research project on the practices and experiences of CHNs and asylum-seeking families in DRC asylum centres, which was a sub-study of a larger Nordic collaborative project on health and integration among refugee youth: Coming of Age in Exile (CAGE).^13^ The analysis was a collective process between all authors.

A.B. obtained verbal informed consent from all participating parents after reading out aloud an information letter, prior to any interview. The letter outlined: our disassociation from the DRC and DIS; the promise to anonymize any identifiable information to maintain the families’ confidentiality; the families’ right to withdraw from the study at any time; and finally our contact details. The Danish Data Protection Agency approved the study, and no formal ethical approval is required for this type of research in Denmark.

### Participants, data collection and analysis

The administrator of the DRC health clinic in the reception centre facilitated contact with three CHNs who worked in regular asylum centres. These CHNs identified families whom A.B. then invited to participate in interviews. The inclusion criteria were that families consisted of at least one care-taker and one child and were in Phase Two of the asylum process. Eleven families agreed to participate in interviews, corresponding to 15 parents in total. Table 1 provides an overview of the families’ characteristics. A semi-structured topic guide was outlined for the interviews, designed to generate insights into the parents’ perspectives, motivations and experiences both with parenting and with CHN consultations. In eight of the interviews, physical and telephone interpreters, employed by the Danish Refugee Council and fluent in the respective language of the informants, were used to mediate the communication between A.B. and the parent(s). The interviews lasted between 40 and 90 min, were audio-recorded and transcribed verbatim.

**Table 1:** Participant characteristics

Interview	Participants	Country of origin	Number of children	Years in Danish asylum system	Number of relocations between asylum centres
Family 1	Father, in-person interpreter	Kuwait (stateless Bedoon)	6	3	3
Family 2	Mother, father, in-person interpreter	Syria	5	4	3
Family 3	Father, physically in-person interpreter	Iraq	3	3	5
Family 4	Mother, physically in-person interpreter	Kuwait (stateless Bedoon)	2	3	6
Family 5	Mother, father, in-person interpreter	Iraq (stateless Kurds)	3	3	4
Family 6	Mother, father, telephone interpreter	Kuwait (stateless Bedoon)	6	3	4
Family 7	Mother (interview in English)	Somalia	2	4	3
Family 8	Mother (interview in Danish)	Jordan	5	5	2
Family 9	Mother, father (interview in English)	Egypt	4	3	5
Family 10	Father, telephone interpreter	Kuwait (stateless Bedoon)	4	2	3
Family 11	Mother, telephone interpreter	Syria	2	2	4

The interview transcriptions were imported into NVivo 12 for thematic coding. The material was analysed thematically inspired by Attride-Stirling’s (2001) Thematic Network Analysis.^14^ This first involved an inductive phase in which the data were organized into 63 codes. For the purpose of this paper, we clustered the codes illuminating the bounded agency of asylum-seeking parents into seven basic themes, which were then clustered into two organizing themes. Table 2 shows the different themes derived in this process.

**Table 2:** Thematic network analysis: from basic themes to global theme

Basic theme	Organizing theme	Global theme
Noise from neighbours Sharing bathroom and kitchen Violence between neighbours Postnatal care is inhibited	**Physical structures** within the asylum centre undermine the parenting efforts of asylum-seeking parents and prevent them from enacting advice and support given by child health nurses	**The bounded agency** of parents in Danish asylum centres
Regular relocations between centres Insufficient money allowance Status as asylum-seekers deprives rights	**Pervasive uncertainty** associated with parents’ asylum status prevents them from providing their children with a safe, healthy and stable environment	

## Results

We identified that the asylum-seeking parents’ capabilities to take care of their children were bounded in two ways, which we illustrate in the following.

### Physical structures: housing, lack of privacy and disturbance

Most parents said that they lacked family privacy as they shared bathroom and kitchen facilities with fellow asylum-seekers, and many stressed how this felt disrespectful to their family structure. Complaints about the physical structures within asylum centres were closely related to the families’ housing conditions. The parents interviewed had experienced housing in various asylum centres, and they explained how these different rooms had been more or less ‘family-friendly’, which for instance related to having one or several rooms, private or shared bathrooms and whether neighbours were families with children, or single men. A father explained that he did not feel capable of protecting and providing safety to his children because of these conditions: ‘If we only want to change a diaper, or one of us wants to use the toilet, it’s a long way, where everyone is watching you (…) this is not what I want for my children’. (Father in Family 2).

He also described how noise from neighbours prevented him and his wife from ensuring their children developed good sleeping habits, which he explained was one of the CHNs’ most important pieces of advice:

The CHN talks a lot about our children’s sleep. It’s so important for them to sleep long enough and well. But how [can they]?—when we live next to a lot of young single men, and many drug and alcohol abusers, who are extremely noisy at night (Father in Family 2).

Other parents had specific experiences where their children had witnessed violence among these ‘noisy neighbours’, which was also a sleep-disturbing factor: ‘One of our neighbours was drunk and in a fight and knocked on our window so hard that it broke. My daughter got so afraid, and had trouble sleeping, and cried even weeks after the incident’. (Mother in Family 5).

Being housed in such ‘family-unfriendly’ asylum centres restricted parents’ ability to fulfil their wishes to be good parents. The mother in Family 7 also experienced her housing inhibiting her postnatal care. After giving birth in a Danish public hospital, her new-born son was losing weight, and while this would usually be an additional concern for new parents, she was relieved as it enabled a longer stay at the hospital with privacy, assistance, safety and rest:

I was deeply grateful that I did not have to go back to the stress. It is stressful for a new and single mother, coming from Somalia, with a new baby, surrounded by noise, having to cook in a shared kitchen and using a shared bathroom (Mother in Family 7).

Housing conditions therefore prevented parents from following all the advice received from CHNs and other DRC employees. Several parents expressed frustration as to why CHNs would encourage parents to ensure good sleeping habits and other aspects of children’s wellbeing, without being able to act when the parents complained about their challenges: ‘We told the CHN, but she doesn’t decide where we live’ (Father in Family 2). Such frustrations exemplify how the CHNs’ supportive care was limited by circumstances, and that they had no influence on DIS’s arrangement of families’ housing.

### Pervasive uncertainty: relocations, money allowance and juridical status

Their juridical status and the organizational conditions during the asylum-seeking process led to pervasive uncertainty amongst asylum-seeking parents. Several parents articulated how the supportive care from CHNs was in contrast to the ‘support’ (provision of basic necessities) provided by the DIS: ‘The CHNs and DRC employees who see us every day are aware of my children’s misery, and that they are not feeling good, but everything in here moves in the opposite direction’. (Father in Family 3). At the time of the interview, Family 3 was about to move to their sixth asylum centre. They were anxious about what awaited them, especially as the new centre was located ‘at the other end of Denmark’. The father further said ‘relocations don’t consider the wellbeing, or rights, of our children’ and explained that he felt incapable of supporting his sons’ mental wellbeing in this process. The parents interviewed had all experienced having to move between different centres, which fostered much distress among both parents and children, making it hard for parents to maintain the best of their parenting skills. Relocations disrupted children’s friendships, caring relations with CHNs and teachers, and also the everyday lives of families and children, which parents had struggled to establish. This meant that families had to ‘adapt in a new centre, with new people and new habits’ (Father in Family 1). One mother similarly explained:

The CHNs encourage us to support our children and their mental health (…) Every time we have to move, I suffer and my children suffer with me. Physically and psychologically. Why don’t they let us stay in *one* centre? My children have lost their friends again and again (Mother in Family 9).

All parents emphasized how these ‘disruptions’ in stability were stressful for them, especially as they led to low spirits among their children. Several parents also said how they had tried to involve the CHNs in advocating for fewer relocations, but that these were decided by the DIS when they closed centres or repurposed, for instance, a family centre into a department centre.

Parents emphasized that CHNs recommended healthy nutrition as an important aspect of structuring children’s everyday life in an asylum centre. Several parents, however, complained about insufficient money allowances, which prevented them from buying groceries for the healthy food that CHNs recommended the parents to cook for their children. The mother in Family 2 told us that the limited money allowance made her breastfeed her 2-year-old son to compensate for his nutritional needs: ‘If I, at least, eat healthily, I assume he’ll get the important nutrients through my milk’. Another mother also described her challenges:

I am in the supermarket and I look at the vegetables the CHN advised me to buy, but when I search my pockets, and I know this money has to cover the next 14 days, it simply isn’t enough and I choose something else (Mother in Family 11).

All parents interviewed believed that an approval of their asylum application would improve their situation on several levels, and especially in relation to the health and wellbeing of their children. The father in Family 3 told: ‘As long as we don’t have residency, our human rights and my children’s rights are not considered’. Having asylum-seeker status was described as putting their children in a liminal position, as they both had child- and human rights, yet were also asylum-seekers without residency. As the father in Family 10 explained: ‘Our children go to school, they are seen by a CHN, and they speak and write in Danish. They learn the Danish values and norms. But if we don’t get residency, none of all that makes sense’. This liminality often left parents in feeling powerless when trying to support their children, as expressed by the mother in Family 5 who told: ‘My children experience their friends in school obtain residency and move to apartments, and they ask us why we still live here. We don’t know what to answer’. Another mother similarly explained:

If my son gets this life-saving treatment, he must undergo surgery every seventh year, but the DIS can’t help as he has no residency [permit], and they [DIS] are afraid we’ll be sent back and the surgery is not available in our country (…) I am afraid, I often cry and feel angry. I don’t want residency. I just want my son to have a good life. The most stressful thing is to look at your child without being able to help him (Mother in Family 8).

This mother described how she felt completely powerless in taking care of her son and providing him safety and health, but her example further illustrated that the status of her son as an asylum-seeker became foregrounded more than his status as a child having equal entitlements to healthcare like children with Danish residency.^15^ This most likely reduced her capacity in safeguarding her son’s, but also her other four children’s, health and wellbeing.

## Discussion

We have elsewhere documented how CHNs manage to establish caring relations with asylum-seeking parents in asylum centres run by the DRC.^10^ In this study, we show that whilst parents appreciate this supportive care, the physical, organizational and juridical structures around asylum centres undermine parents’ capacities to act on advice from CHNs, and to follow their own desires as parents. Our findings build on previous research. A synthesis of 138 international qualitative studies (of which 10 specifically focus on asylum-seekers) concludes that asylum-seeking parents’ capacities to support their children are challenged by uncertainty about their future and fears of their asylum claim being rejected.^2^ The families in our study had been in the asylum system for up to 6 years, and several studies note that waiting time, and frequent relocations, add to uncertainty ‘and’ to family-breakdown among asylum-seekers.^7^^,^^16–19^ Housing and poverty constitute major constraints in this study, which echoes findings from Irish and Belgian asylum centres. Similarly with our findings, these note that lack of family privacy, violence, financial challenges, tight time schedules and being on food allowance cause isolation, disempowerment, loss of control over own children and over parenting methods among asylum-seeking parents.^8^^,^^20^^,^^21^

Whereas Evans (2007) suggests that the constraints constituting the bounded agency of individuals can sometimes be moved, circumnavigated, altered or resisted,^11^ we argue that in the case of asylum seekers, the agency of these parents is ‘tightly’ bounded, very constrained, as they have very few opportunities to influence their circumstances. A sole example is the mother who, because of her unique situation, compensated for her son’s need for nutrients by breastfeeding him more frequently, and thereby circumnavigated the undermining effect of insufficient money allowance. Our focus on the bounded agency of the parents interviewed, also reveals ‘inevitable contradictions’ within the asylum system: as humanitarian employees, CHNs explicitly aim to support parents in taking care of their children, and to make the asylum-seeking period more ‘tolerable’ for families^22^; however, these efforts collide with politically decided physical, organizational and juridical structures that frame the everyday lives of asylum-seekers. Thus, the housing, relocations and money allowances of asylum-seeking parents, all arranged by the DIS, undermine their parental agency to use and act on support and advice from CHNs. Therefore, CHNs’ encouragements to be autonomous, and CHNs’ provision of advice to parents on creating a healthy environment for their children, only feels supportive during specific consultations with CHNs, but when families leave the CHNs, they are limited by their environment from benefitting entirely from this support.^10^ We acknowledge that information provided by CHNs is, in general, rarely fully implemented in family practices because of difficult circumstances^23^; however, in the context of asylum-seekers, there are particular tightly binding, politically decided structures, over which families have no influence.

Highlighting the inevitable contradictions is not to state that the humanitarian efforts of CHNs are unnecessary: the CHNs’ supportive care may indeed compensate for some of the uncertainty otherwise pervasive for parents who live in an asylum centre. Rather, we seek to highlight a need for explicit attention to be paid to the parenting roles and capabilities of asylum-seekers, to reconsider their physical and organizational environments, and to create safe, stable and healthy homes for the parents and their children. Our findings have several implications for future public health policy, research and practice. We call for heightened attention towards improving family-friendliness within reception facilities across Europe, as well as minimizing relocations between different centres. Belgian and Bulgarian scholars warn that housing in asylum centres reduces parents’ autonomy and abilities to create a home, and instead isolates and marginalizes families from being part of a community.^1^^,^^8^ This is disconcerting, as migration scholars from the Netherlands and the UK emphasize that asylum-seeking families especially need to feel they belong, and that they can reconstitute a family-functioning environment, establishing a new home in the destination country.^24^^,^^25^ Parents’ autonomy and agency to ensure their children a healthy, stable and supportive environment is important in all contexts and periods of life.^1^ Whereas several parents in our study expressed that a residency permit would improve their situation, a Swedish study found other and new difficulties related to parenting experiences among ‘refugee’ parents even after successfully obtaining Swedish residency (e.g. having only temporary residency, learning the language, finding a job, living in temporary housing, waiting for family members to be reunified and feeling lonely).^26^

This calls for a heightened focus on parental agency across several phases of migration trajectories, within future public health research and practice. Further insight into the emotional and psychological reactions of parents to their bounded parental agency is also needed. Our findings indicate that the wellbeing of both parents and their children is affected negatively, adding to the solid body of European literature documenting challenges to the mental health and wellbeing of both asylum-seeking children and adults.^27–29^ Having active agency (parental and otherwise) has been framed as a protective factor against mental health problems among adult asylum-seekers,^30^ which supports our call for specific focus on and action to alleviate the bounded agency of asylum-seeking parents, and thereby improve the wellbeing of families through a safe, stable and healthy environment.

There are limitations that should be considered when interpreting our results. All families were awaiting a second decision of their asylum application from the Refugee Appeals Board, which could have negatively affected their accounts. However, based on the parents’ positivity towards the CHNs, we believe this is a minor issue. The necessity of interpreters to mediate all communication between interviewer and interviewees will always mean that details may get lost in translation, which should be seen as a limitation to the depth of our insights.^31^ However, the background of A.B. (she was pregnant and had a different ethnic background than Danish) was noted to foster parents’ engagement during interviews, which is a strength as others have described the challenges of engaging asylum-seekers in research.^32^ In relation to this, we used both telephone and in-person interpreters. Telephone interpreters had fewer opportunities to engage with our informants compared with the in-person interpreters, increasing the risk of misunderstandings. However, A.B. experienced that in-person interpreters could undermine the relation between herself and the families through interference.

Our findings suggest that whereas CHNs do their best to make the asylum process more tolerable for families by engaging in caring relationships with parents, these parents are constrained in performing some of the fundamental tasks related to parenting. Asylum-seeking parents are effectively disempowered in their parenting role. They are for instance limited in choosing where to live, having an adequate income, buying healthy foods, having privacy as a family and having the power to maintain a safe, stable and healthy environment for their children. Asylum-seeking parents’ bounded agency most likely contributes to mental distress, which also risks having an impact on the mental health and wellbeing of children. There is an urgent need to re-think the asylum-seeking process, and to put in place the systems and structures that empower asylum-seeking parents to be the parents they want to be, and parents who are able to take heed of advice and support given by CHNs. In the meantime, we have elsewhere documented the tactics CHNs adopt—in the context of these constraints—to provide care and advice that resonate with the families’ current life situations.^10^
